# The Ability of Bile to Scavenge Superoxide Radicals and Pigment Gallstone Formation in Guinea Pigs

**DOI:** 10.1155/1996/20687

**Published:** 1996

**Authors:** Cong Lin, Tao Shen, Xianbo Fu, Xiaosi Zhou

**Affiliations:** Department of Surgery Third Teaching Hospital Beijing Medical University Beijing 100083 China

## Abstract

After partial ligation of the common bile duct (CBD) of guinea pigs, 14 of 16 animals developed pigment gallstones within one week (S group). Intraperitoneal injection of Vit. E and C, each 10 mg/kg daily from 3 days before CBD ligation to one week after the operation (S+V group), decreased the gallstone incidence to 5/14 (exact probability<0.01). The gallstone incidence in the control group, that only received laparotomy without ligation of the CBD, was 0/15. Biochemical analysis of the gallbladder bile showed that stricture of the CBD was associated with a significant increase in levels of unconjugated bilirubin (UCB) and Ca^2+^ (*p*<0.05 and <0.01). Simultaneously the scavenging rate (SR) of superoxide radical in bile significantly decreased (*p*<0.05). Comparing S+V group with S group, the effect of Vit. E and C on the concentrations of UCB and Ca^2+^ in bile was not significant (both *p*>0.05), but Vit. E and C normalized the SR, and the difference between S group and S+V group was significant (*p*<0.05). These results suggested that Vit. E and C, known as antioxidants, enhanced the ability to scavenge oxygen radical in S+V group; and that in addition to the increases of UCB and Ca^2+^ concentrations, the participation of oxygen radicals might be of importance for pigment gallstone formation induced by bile duct obstruction.


*HPB Surgery*, 1996, Vol.10, pp. 73–77
Reprints available directly from the publisher
Photocopying permitted by license only

© 1996 OPA (Overseas Publishers Association)
Amsterdam B.V. Published in The Netherlands
by Harwood Academic Publishers
Printed in Malaysia

### The Ability of Bile to Scavenge Superoxide Radicals and Pigment Gallstone Formation in Guinea Pigs*

CONG LIN, TAO SHEN, XIANBO FU and XIAOSI ZHOU

Department of Surgery, Third Teaching Hospital, Beijing Medical University,
Beijing, 100083, P. R. China

*(Received 10 February 1994)*

After partial ligation of the common bile duct (CBD) of guinea pigs, 14 of 16 animals developed pigment gallstones within one week (S group). Intraperitoneal injection of Vit. E and C, each 10 mg/kg daily from 3 days before CBD ligation to one week after the operation (S+V group), decreased the gallstone incidence to 5/14 (exact probability<0.01). The gallstone incidence in the control group, that only received laparotomy without ligation of the CBD, was 0/15. Biochemical analysis of the gallbladder bile showed that stricture of the CBD was associated with a significant increase in levels of unconjugated bilirubin (UCB) and $Ca^{2+}$ ($p<0.05$ and $<0.01$). Simultaneously the scavenging rate (SR) of superoxide radical in bile significantly decreased ($p<0.05$). Comparing S+V group with S group, the effect of Vit. E and C on the concentrations of UCB and $Ca^{2+}$ in bile was not significant (both $p>0.05$), but Vit. E and C normalized the SR, and the difference between S group and S+V group was significant ($p<0.05$). These results suggested that Vit. E and C, known as antioxidants, enhanced the ability to scavenge oxygen radical in S+V group; and that in addition to the increases of UCB and $Ca^{2+}$ concentrations, the participation of oxygen radicals might be of importance for pigment gallstone formation induced by bile duct obstruction.

KEY WORDS: Free radical pigment gallstone formation

### INTRODUCTION

Free radical signals have been detected in pigment gallstones (PS) in vivo under anoxic condition, and the intensities of the signals correlated positively with the amounts of calcium bilirubinate[1]. However, obstruction of the biliary tract could induce an increase of both free radical reaction[2] and formation of PS[3]. These results suggested that oxygen radicals might be related to the formation of PS. The aim of this experiment is to study the relation between active oxygen radicals and PS formation using a guinea pig model, in which PS were induced by partial ligation of common bile duct (CBD).

*This work was supported by the National Natural Science Foundation of China, No. 3880768.
*Correspondence to*: Dr. X. S. Zhou, Department of Surgery, Third Teaching Hospital, Beijing Medical University, 49 North Garden Road, Beijing, 100083, P. R. China.

### MATERIALS AND METHODS

### *Reagents*

Bilirubin (99%) was purchased from fluka Company (Switzerland). Xanthine oxidase was purchased from Dongfend Biochemical Technical Company of Shanghai Biochemical Institute (Chinese Academy of Sciences). Xanthine was obtained from Sigma Chemical Company (USA).

### *Grouping of Animals*

52 male guinea pigs (300–700 g body weight) were divided into three groups. (1) Stricture group (S): 19 animals, their CBD were partially ligated[3]. Normal saline was intraperitoneally administered as placebo from 3 days before the operation to 7 days after the operation. (2) Stricture plus Vitamin E, C group (S+V): 18 animals, the operation was the same as that of S group, but the normal saline was substituted with vitamin E and C, each 10 mg per Kg body weight, injected intraperitoneally once a day. (3) Control group (C), 15 animals, laparotomy without ligation of CBD was performed, and the use of the placebo is the same as S group.

*Gallstone Incidence and Bile Analyses*

The guinea pigs were sacrificed 7 days after the operation.Having fasted for 12 hours, animals were anesthetized by intraperitoneal injection of sodium pentobarbital (30 mg/Kg body weight). Laparotomy was performed. Gallbladder bile was collected and gallstones in the biliary tree were observed. After 1100 g centrifugation for 15 minutes, the scavenging rate (SR)[4,5] of bile on exogenous superoxide radical ($O_2^{\cdot}$), concentrations of ionized calcium (ICa), total bilirubin (TBr) and indirect reactive bilirubin (IBr)[6], which represents unconjugated bilirubin (UCB), in the supernatant were determined.

*Statistical Analyses*

For the comparison of gallstone incidence, exact probability was calculated. The results of gallbladder bile components was expressed as mean ± standard deviation, and grouped Student's t-test was used. *P* values less than 0.05 were accepted as significant.

### RESULTS

*Gallstone Incidence*

Three animals in S group died of complete CBD ligation on the fourth day after the operation, and gallstones were found in all of them. In S+V group, four animals died ahead of schedule and gallstones were not found in them. Two of them died of complete CBD ligation (four and five days after operation respectively); the other two did of rupture of abdominal incision on the sixth day. These seven animals were excluded.

The PS incidence of S group (14/16=87.5%) and S+V group (5/14=35.7%) were significantly higher than that of C group (0/15, $p$<0.01 and $p$<0.05 respectively); but the PS incidence of S+V group was significantly lower than that of S group ($p$<0.01) (see Figure).

*Gallbladder Bile Components*

In some animals, their gallbladder bile was too small to satisfy the need of biochemical analysis. In this case, bile specimens from two individuals in a same group were mixed up and used, even so, some mixed bile specimens were still not enough for all the bile component items. Therefore , the numbers of bile specimen, "n" in the table for each bile component, were not the same and often less than the numbers of animals allocated in the corresponding group, especially in the C group (see table).

The $Ca^{2+}$, TBr and IBr concentration of gallbladder bile in S group were significantly higher than those of C group ($p$<0.01, 0.05, and 0.05 respectively); and the SR decreased significantly ($p$<0.05). In S+V group, the $Ca^{2+}$ and IBr concentrations were not significantly different from those of S group ($p$>0.05 both), and the concentration of TBr was even higher ($p$<0.05). But, SR rose significantly from the level of S group ($p$<0.05), and closed to that of C group ($p$>0.05) (table and figure).

### DISCUSSION

Our previous study[6] revealed that the increase of either UCB or $Ca^{2+}$ concentration would elevate the ion product of [UCB] and [$Ca^{2+}$]. Once the ion product exceed their conditional solubility product (K'sp), calcium-bilirubinate would precipitate. This is called EQUILIBRIUM THEORY of calcium bilirubinate precipitation and dissolution.

**Table 1** Gallbladder bile components

| *Bile components* | | *Group* | | | *t-test* | |
|---|---|---|---|---|---|---|
| | | *S* | *S+V* | *C* | *S vs C* | *S+V vs S* |
| ICa | mean | 765 | 720 | 281 | | |
| uM | SD | 441 | 304 | 113 | t=3.537 | t=0.315 |
| | n | 16 | 14 | 11 | $p$<0. 01 | NS |
| TBr | mean | 40.8 | 69.2 | 13.7 | | |
| uM | SD | 30.3 | 40.3 | 8.1 | t=2.301 | t=2.165 |
| | n | 16 | 13 | 7 | $p$<0. 05 | $p$<0. 05 |
| IBr | mean | 12.6 | 16.9 | 5.2 | | |
| uM | SD | 8.9 | 11.5 | 3.4 | t=2.106 | t=1.124 |
| | n | 16 | 13 | 7 | $p$<0. 05 | NS |
| SR | mean | 68.3 | 92.1 | 94.4 | | |
| % | SD | 31.1 | 8.2 | 4.8 | t=2.475 | t=2.772 |
| | n | 14 | 14 | 9 | $p$<0. 05 | $p$<0. 05 |

note: C = Control group S = Stricture group
S+V = Stricture plus Vit. E, C group
ICa = Ionized calcium TBr = Total bilirubin
IBr = Unconjugated bilirubin SR = Scavenging rate
NS = No significance

Accordingly, the rise of PS incidence from C group to S group in this study might be explained by the coincident significant elevation of UCB and $Ca^{2+}$ concentrations in bile. But, the drop of PS incidence from S group to S+V group might not be attributed to the coexistent change of the UCB and $Ca^{2+}$ concentrations in bile, because the decrease of $Ca^{2+}$ concentration was not significant; furthermore, the UCB concentration of S+V group even was slightly higher than S group. On the other hand, it is striking that when the PS incidence rose from C group to S group, the SR decreased significantly; when the PS incidence dropped from S group to S+V group, the SR increased significantly and approximated to the level of C group. These facts suggested that in addition to the increase of UCB and $Ca^{2+}$ concentrations, the participation of free radical might be an essential factor in the PS formation. But, within the S+V group between animals who developed PS and those who did not, the difference in SR (89.0±8.8, $n$=5 vs 93.8±7.8, $n$=9) was not significant ($p$>0.05). This suggested that factors other than SR and the equilibrium of calcium bilirubinate might exist, and this is to be investigated.

The necessity of free radicals in PS formation does not belittle the importance of equilibrium theory. Because, the experiments in vitro of our colleagues[7] revealed that there were three effects of free radical on the interaction of calcium ion and bilirubin in the presence of sodium cholate : (1) accelerating the speed of precipitation; (2) decreasing the K'sp; and (3) enlarging the size of precipitated calcium bilirubinate particles. All of these effects promoted the precipitation and polymerization of calcium bilirubinate. Their findings in vitro and the results form this experiment in vivo supported each other. The findings also revealed that free radical changed the equilibrium condition under given concentrations of [UCB] and [$Ca^{2+}$], such as the reaction speed, the K'sp, and the particle size of precipitate. The basis of the free radical effectiveness is the precipitation and dissolution equilibrium itself.

In addition, oxygen radicals may promote the formation of gallstones by stimulating glycoprotein secretion[8]. The conditions for the stricture group and control group in Xu's experiment were the same as those of S group and C group in this experiment respectively. He had determined the content of glycoprotein of gallbladder bile. The glycoprotein content of stricture group was significantly higher than that of control group[3]. The important role of glycoprotein in the formation of both pigment and cholesterol stones is well known. Consequently, the PS incidence of S+V group being lower than that of S group might be related to inhibiting the glycoprotein secretion by scavenging oxygen radicals.

### CONCLUSION

1. The incomplete obstruction of guinea pig common bile ducts resulted in high pigment gallstone incidence. At the same time, the ability of scavenging

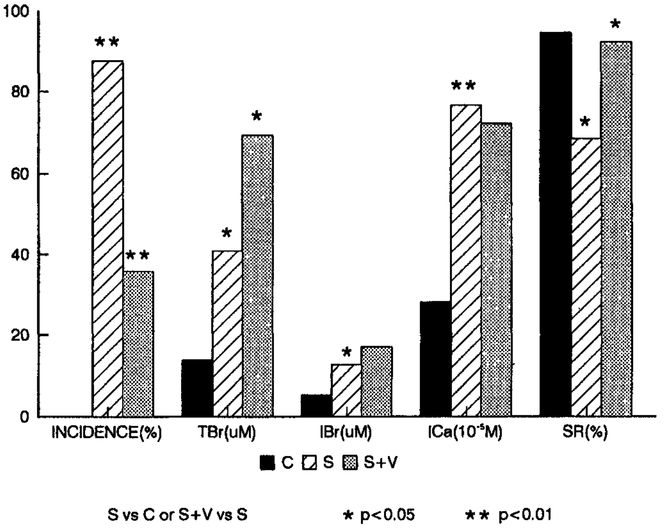

**Figure1** The relationship between pigment gallstone formation and gallbladder bile components with bile duct obstruction. Comparison of the gallstone incidence, the scavenging rate (SR), and the concentrations of total bilirubin (TBr), indirect reactive bilirubin⁻ (IBr), and ionized calcium (ICa), of gallbladder bile in control (C), stricture (S), and stricture plus Vit. E, C (S+V) groups.

exogenous $O_2^{\cdot-}$ in bile decreased, the UCB and $Ca^{2+}$ concentrations of gallbladder bile increased.

2. Administration of Vit. E, C to the animal models recovered the scavenging rate of the gallbladder bile on exogenous $O_2^{\cdot-}$ to the level of control; in spite of that UCB and $Ca^{2+}$ concentrations remained high, the incidence of PS decreased significantly. This showed that in addition to the concentrations of UCB and $Ca^{2+}$, free radical is another essential factor in PS formation.

3. Although administration of Vit. E and Vit. C was capable of decreasing the incidence of pigment gallstone by enhancing the ability of scavenging oxygen radicals in bile, it was not capable to preventing pigment gallstone formation thoroughly.

### REFERENCES

1. Shen, T., Lin, C., Fu, X.B., Zhou. X.S. (1992) Free radical appears in gallstones in vivo. *HPB Surgery.*, 5 (suppl): 102.
2. Yang, K.Z., Zhang, J.C., Yin, G.Q., Wang, J.W., Li, K. (1989) Superoxide radical reaction in bile and gallstone. Chinese Medical Association: Proceeding of the Fourth National Conference of Biliary Surgery, *Cheng Du, Oct.*, 1.
3. Xu, Z., Zhang, W.H., Fu, X.B., Shao, X.M., Zhou, X.S. (1990) Changes of bile components during the formation and prevention of black pigment gallstone in a guinea pig model. *Chinese Journal of Surgery.*, **28**, 558–561.
4. Elstner, E.F., Heupel, A. (1976) Inhibition of nitrite formation from hydroxylammonium chloride: a simple assay for superoxide dismutase. *Anal Biochem.*, **70**, 616–620.
5. Oyanagui, Y. (1984) Reevaluation of assay methods and establishment of kit for superoxide dismutase activity. *Anal Biochem.*, **142**, 290–296.
6. Fu, X.B., Zhou, X.S., Zhange, W.H., Deng, S.Q., Shao, X.M. (1985) "Bilirubin-calcium compound" precipitation and the effect of bile salts on it. *Chin Med J.*, **98**, 728–738.
7. Liu, X.T., Tang, B., Wang, K. (1990) A study of the interaction of calcium ion and free radical treated bilirubin in the presence of sodium cholate. *J Beijing Med Univ.*, **22**, 285–286.
8. Hale, W.B., Turner, B., Lamont, J.T. (1987) Oxygen radicals stimulate guinea pig gallbladder glycoprotein secretion in vitro. *Am J Physiol.*, 253 (Gastrointest Liver Physiol. 16): G627–630.

### COMMENTARY

Pigment gallstones are of two major types, black and brown stones. These two pigment types stones are morphologically, compositionally and clinically distinct[1,2].

Black pigment gallstones forms usually within the gallbladder although they can migrate to the bile duct[1]. They are associated with heamolytic anaemia, cirrhosis, and alcoholism. The incidence of black gallstone disease increases with age[2]. The pathogenesis of black stones is probably related to nonbacterial, nonenzymatic hydrolysis of bilirubin conjugates, especially the monoconjugates[3].

De novo common bile duct stones are likely to be brown pigment stones. The presence of a juxtapapillary duodenal diverticulum is an important associated risk factor for formation of brown pigment stones probably because of the change in the bacterial flora in the region of the papilla[4]. Brown pigment stones are formed with stasis or infection in the biliary system[1]. Maki postulated that such stones form following hydrolysis of bilirubin diglucuronide by bacterial glucuronidase to form glucuronic acid and bilirubin[5]. Unlike bilirubin diglucuronide, which is freely water soluble, unconjugated bilirubin is almost insoluble in water and may precipitate by combining with calcium to form calcium bilirubinate[1]. Both bacteria[5] and tissues[6] contain glucuronidases, and although the pH optimal for the enzymes are different, the pH of the microenvironment in which the hydrolysis occurs is unknown and may allow either or both types of enzymes to act[1]. Similarly both bacteria and tissue contain phospholipases[7] and the fatty acids present in the stone are very likely the product of either bacterial or tissue catalysis or hydrolysis of biliary lecithin[8]. Recent data indicate that a variety of calcium salts of fatty acids are present in brown stones. Palmitate is only one of these salts[2].

This study attempts to show that oxygen radicals might be related to the formation of pigment stones in animals with partial bile duct occlusion in addition to the increases in unconjugated bilirubin and calcium concentrations. The administration of intraperitoneal vitamins E, C, known as antioxidants, is capable of decreasing the incidence of pigment stones but not completely preventing its formation, suggesting that free radicals may play a role in pigment stone formation.

W.Y. Lau, F.R.A.C.S.
A.K.C. Li, F.R.A.C.S.
Department of Surgery
The Chinese University of Hong Kong
Prince of Wales Hospital
Shatin, New Territories, Hong Kong

### REFERENCES

1. Soloway, R.D., Trotman, B.W., Maddrey, W.C. and Nakayama F. (1986) Pigment gallstone composition in patients with hemolysis or infection/stasis. *Dig Dis Sci.*, **31**, 451–460.
2. Trotman, B.W. (1991) Pigment gallstone disease. *Gastroentrol Clin N Am.*, **20**, 111–126.

3. Spivak, W., diVenuto, D., and Yuey W. (1987) Non-enzymatic hydrolysis of bilirubin mono- and diglu curonide to unconjugated bilirubin in model and native bile systems. *Biochem J.*, **242**, 323–329.
4. Skar, V., Skar, A.G., and Osense M. (1989) The duodenal bacterial flora in the region of pailla of Vater in patients with and without duodenal diventicula. *Scad J Gastroenterol.*, **24**, 649–656.
5. Maki, T. (1966) Pathogenesis of calcium bilirubinate gallstone: Role of E. coli, glucuronidase and coagulation by inorganic ions, polyelectrolytes and agitation. *Ann Surg.*, **164**, 90–100.
6. Musa, B.V., Doe, R.P., and Seal, U.S. (1965) Purification and properties of human liver beta-glucronidase. *J Biol Chem*, **240**, 2811–2816.
7. Sjodahl, R., Tagesson, C. (1976) On the medication of the inflammatory reaction in the human gallbladder epithelium. *Scand Gastroenterol.*, **11**, 321–328.
8. Robins, S.J., Fajulo, J.M., Patton, G.M. (1982) Lipids of pig ment gallstones. *Biochem Biophys. Acta.*, **712**, 21–25.

